# Online-group intervention after suicide bereavement through the use of webinars: study protocol for a randomized controlled trial

**DOI:** 10.1186/s13063-019-3891-5

**Published:** 2020-01-08

**Authors:** Birgit Wagner, Laura Hofmann, Ulrike Maaß

**Affiliations:** 1 Medical School Berlin, Calandrellistraße 1-9, 12247 Berlin, Germany; 2 Potsdam University, Karl-Liebknecht-Str. 24-25, 14476 Potsdam, Germany

**Keywords:** Suicide bereavement, Grief, Group intervention, Webinar, Suicidality, Prolonged grief disorder, Randomized controlled trial

## Abstract

**Introduction:**

The death of a significant person through suicide is a very difficult experience and can have long-term impact on an individual’s psychosocial and physical functioning. However, there are only few studies that have examined the effects of interventions in suicide survivors. In the present study, we examine an online-group intervention for people bereaved by suicide using a group-webinar.

**Methods:**

The intervention was developed based on focus groups with the target group. The cognitive-behavioral 12-module webinar-based group intervention focuses on suicide bereavement-related themes such as feelings of guilt, stigmatization, meaning reconstruction and the relationship to the deceased. Further, the webinar includes testimonial videos and psychoeducation. The suicide survivors are randomized to the intervention or the waiting list in a group-cluster randomized controlled trial. Primary outcomes are suicidality (Beck Scale for Suicide Ideation) and depression (Beck Depression Inventory-II) and secondary outcomes are symptoms of prolonged grief disorder (Inventory of Complicated Grief-German Version ), posttraumatic stress disorder ( Revised Impact of Event Scale ), stigmatization (Stigma of Suicide and Suicide Survivor ) and posttraumatic cognitions (Posttraumatic Cognitions Inventory).

**Discussion:**

Previous studies of Internet-based interventions for the bereaved were based on writing interventions showing large treatment effects. Little is known about the use of webinars as group interventions. Advantages and challenges of this novel approach of psychological interventions will be discussed.

**Trial registration:**

German Clinical Trials Register, DRKS00014426. Registered on 12 April 2018.

**Protocol Version:**

3, 21.10.2019.

## Introduction

### Background and rationale

Global data showed that age-standardized mortality rates for suicide decreased by 32% worldwide between 1990 and 2016 [1]. However, suicide is still the leading cause of age-standardized years of lost life in the global burden of disease, specifically in high-income countries. The suicide of a significant person can leave a long-term impact on the surviving parent, child, partner, sibling or friend. While past research estimated about six individuals are affected by suicide [2], more recent studies reported much higher estimates, including not only the nuclear family, but also taking into account the social network of the deceased person. Berman [3] estimated that 45–60 people are intimately affected by suicide death, depending on the close daily or weekly contact the deceased person had with their social network.

Since 1989, the World Health Organization (WHO) has described people bereaved through suicide as a high-risk group for suicides and describes the aftercare of this group of victims as an essential part of national and international suicide prevention [4]. Suicide bereavement differs significantly in the grieving process from other circumstances of death. Typical reactions after a suicide are, in addition to normal grief reactions, the feeling of guilt and responsibility for the suicide, feeling of shame and rejection, low self-esteem, one’s own suicidal tendencies, the experience of stigmatization, and exposure to traumatic images [5]. Those affected often suffer lifelong functional impairment (e.g. inability to work, early retirement) as a result of suicide [6]. Kersting and colleagues [7] associated a higher risk of prolonged grief disorder in people bereaved by suicide (18.1%) or violent death (20%) compared with people responding to sudden but natural deaths. Furthermore, people bereaved by suicide report sleep disorders, somatic diseases (e.g. stomach ache, headache), and a significantly increased risk of being hospitalized [5, 8]. They are also more susceptible to crises [9]. In fact, more than half of the bereaved suffer depression and about 20% alcoholism, with the combination of the different disorders in particular having the highest suicide risk [10–14]. Pitman and colleagues [5], in their systematic review on mental health outcomes after suicide bereavement, noted increased admission to psychiatric care specifically in bereaved parents after a child’s death through suicide and an increased risk of depression in bereaved children after parental suicide.

Further, numerous population-based representative studies have demonstrated higher mortality after suicide compared to control groups. A Swedish epidemiological study of bereaved siblings (*N* = 160.588) showed that the brother or sister’s suicide in all age groups (18–69 years) had an up to 3.1 times higher mortality rate among the siblings than in non-sibling mourning control groups [15, 16]. Guldin and colleagues [17] investigated the consequences of the death of a parent in the first 18 years of life related to the suicidality of adult offspring (*N* = 7.302.033). The adult children had an 82% higher risk of suicidal death if a parent also committed suicide. Children who had a parent who died before they reached 6 years of age were specifically at higher risk to commit suicide themselves, and this risk remained high for at least 25 years [17]. Therefore, the suicide of a significant person does not only increase the risk of mental health disorders in the bereaved but they themselves also have an increased risk of death by suicide. For this reason, bereavement interventions aimed specifically at people who are bereaved by suicide play an important role in the aftercare.

### Interventions for suicide survivors

Interventions targeted at people bereaved by suicide are an important measure in the prevention of psychiatric illnesses and suicidal behavior. While self-help groups or counseling services for suicide survivors in the voluntary sector steadily increase, only a few psychotherapeutic interventions for survivors following suicide have been developed and have been scientifically evaluated. A recent systematic review included a total of 11 studies aimed at suicide bereavement; however, only a few of the included studies showed evidence of effectiveness in people suffering uncomplicated grief, and empirical evidence for interventions aimed at prolonged grief disorder after suicide is still lacking [18]. McDaid and colleagues [19], in their systematic review, point to the lack of methodological quality of these studies, and thus, a lack of evidence-based interventions. The thin empirical evidence available, however, suggests that (cognitive-behavioral) interventions might be effective for the reduction of maladaptive grief reactions and the perceived responsibility for suicide [20], the reduction of anxiety and depression in suicidal children [21], and the reduction of suicide-associated grief and depression [22]. In summary, there is a great need for evidence-based psychological interventions for suicide survivors.

### Internet-based bereavement intervention

In recent years, internet-based psychological support has been established in social media for bereaved populations, including online support groups (e.g. Facebook), memorial websites and discussion forums. Parallel to the use of social media, web-based interventions for different types of losses have been developed and evaluated in recent years. These Internet-based bereavement interventions were aimed at bereaved people in general [23–26] or specific groups of bereaved people such as parents who have experienced loss of pregnancy [27, 28]. The treatment effects of Internet-based interventions for grief symptoms were moderate to large, and could be maintained over time [29]. The largest and most robust effects, however, were on grief-related symptoms of PTSD. Most Internet-based bereavement interventions included writing assignments, ranging from extensive structured writing assignments to online diaries and homework assignments, and were based in an individual setting.

Unfortunately, even though they are a high-risk group, suicide survivors are precisely the group that is least likely to seek professional help. A study from the UK showed that suicide survivors in particular were less likely to receive formal (e.g. psychotherapists, doctors) or informal support (e.g. friends, religious counseling), and only every fourth interviewed person made use of any form of support at all. Reasons for the low use of professional psychological support include, for example, a lack of psychosocial care or fear of stigmatization (Pitman et al., 2016b; Pitman et al., 2017). Therefore, the anonymity of the Internet might support bereaved patients in overcoming their initial shame or perceived feelings of stigma, which might prevent them from looking for support. Another advantage of Internet-based intervention is that these interventions offer geographic independence and widespread dissemination of the offer of treatment. This is of particular interest for people living in remote areas without any access to local bereavement groups or specialized grief counsellors. Further, Internet-based interventions provide a more user-friendly and flexible approach that is more responsive to a mobile and digitalized society.

The aim of this study is to develop and evaluate a manualized, CBT-based, online, group intervention after suicide bereavement, using webinars. Previous Internet-based bereavement interventions were almost always writing interventions based on an individual setting. Contrarily, we chose the novel format of a webinar to provide real-time simultaneous interaction between the therapists and the group participants. Grief group interventions can provide unique therapeutic elements such as universality of the suffering, group cohesiveness, role model learning, and interpersonal learning [30, 31]. So far, studies of online group interventions have taken place mainly via videoconferencing. Backhaus and colleagues [32], in their systematic review of videoconferences in psychotherapy, included seven studies altogether that used the group format. The results indicated similar clinical outcomes such as face-to-face interventions. Webinars are, next to videoconferencing, most similar to traditional group interventions for people bereaved by suicide. Webinars provide the possibility to participate in group sessions from a remote location in real time but have the additional advantage of providing the possibility of showing videos and psycho-educative PowerPoint presentations, as is customary in e-learning environments.

### Objectives

The main objective of this trial and the proposed online-group intervention is to reduce depression and suicidality in people bereaved by suicide. Secondary outcomes of the trial are prolonged grief disorder, posttraumatic stress disorder, anxiety, stigmatization, posttraumatic grief cognitions and social support. More precisely, our treatment-specific objectives are to determine whether:

1. The online group intervention significantly reduces symptoms of depression, suicidality, prolonged grief disorder, posttraumatic stress disorder, and anxiety after bereavement through suicide.
2. The treatment effects (symptom reduction of primary and secondary outcomes) of the online group intervention are significantly greater than those of the control group after the end of treatment.

In addition, the study addresses the following process-specific research questions:

1. Which factors (such as age, sex, depression, severity of symptoms) are most likely to predict the effect of the treatment?
2. To which extent does the relationship with the deceased (e.g. spouse, sibling, child, parent, friend) influence treatment outcomes in terms of grief and trauma symptoms?
3. Which cognitive processes (such as stigma experience, guilt) influence the general psychopathology of study participants (e.g. depression, prolonged grief, trauma symptoms)?

### Trial design

This study is a randomized control group design with two groups of equal sample size: the treatment group and the waitlist control group. The treatment group will receive treatment through the online group intervention immediately after registration. As a result of the focus groups with suicide survivors, the waitlist control condition was selected as the only one acceptable to the suicide survivors involved. A control that would not receive the intervention after the waiting period could reduce the motivation to participate.

This intervention is based on cognitive behavioral principles (psychoeducation, cognitive restructuring, dealing with difficult emotions such as guilt, shame, suicidality) and will take place once a week over a course of 12 weeks in total. The waitlist control group will receive the same intervention 12 weeks after registration. Participants will be randomly assigned to either group using computer-generated block-randomization. The study protocol was written in accordance with Statement Standard Protocol Items: Recommendations for Interventional Trials (SPIRIT) 2013 [33]; for the SPIRIT Checklist see Additional file 1).

## Method

### Study setting and recruitment

The main recruitment centers are located in Berlin, Leipzig, and Bayreuth, and are directed in accordance with the Medical School Berlin, the Federal Association of Bereaved Parents and Grieving Siblings in Germany (VEID e.V.), and the Association for Relatives to Suicide (AGUS e.V.). In addition, we will recruit participants through the study website (www.hilfe-nach-suizid.de) and advertisements in social media (e.g. social media platforms, self-help forums, specific websites for bereaved people), newspaper articles, flyers for self-help groups, and churches.

### Participants and eligibility criteria

All participants who meet the following criteria will be included in the study: (1) aged between 18 and 75 years, (2) experienced the loss of a close person through suicide, (3) have access to the Internet, (4) have sufficient German language skills, and (5) provide a signed consent form (Additional file 2). Participants with any of the following criteria will be excluded from the study: (1) acute suicidality, (2) elevated depression (defined by a score > 35 on the Beck Depression Inventory-II ), (3) bipolar disorder, (4) current psychotic experience, (5) elevated alcohol, drug, or substance use, (6) suspected borderline personality disorder (BPD), or (7) self-injurious behavior. The group intervention will be conducted by two group leaders: (1) an approved psychotherapist or a trainee in psychotherapy in the last phase of the training and (2) a group leader of a face-to-face self-help group for suicide bereavement.

### Sample size

We based the power and sample size estimations in the current study on results from previous studies examining the effectiveness of online bereavement interventions [34]. Assuming a between-subject effect size of *d* = 0.80, power of 0.80, alpha of 0.05 (two-sided), and a dropout rate of 30%, the within-group sample size should be at least 52 participants. Hence, we aim to include 104 participants in total.

### Procedure

**Fig. 1:**
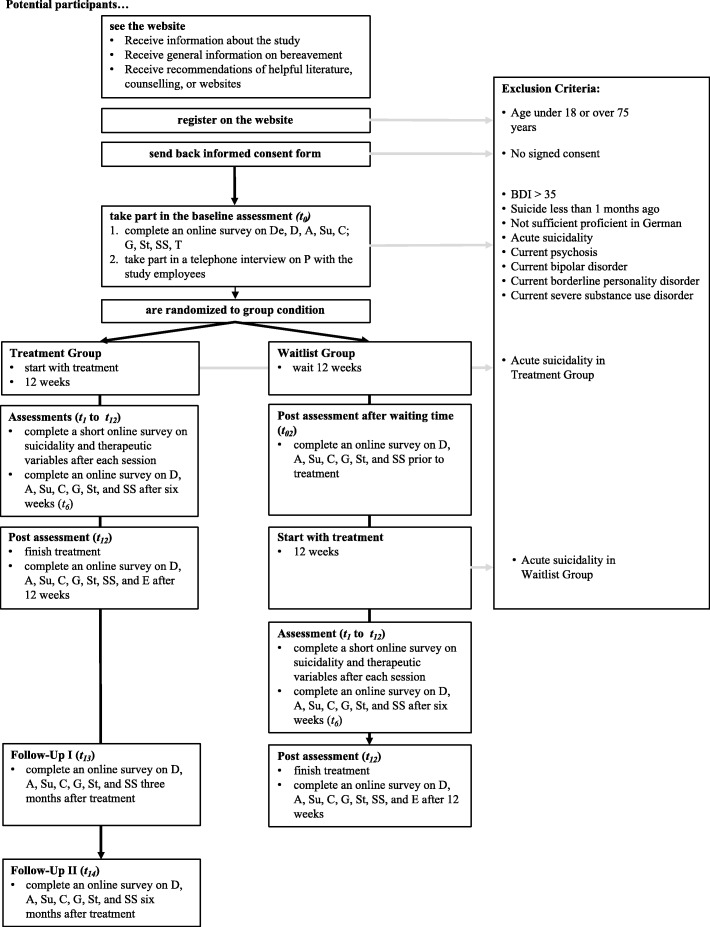
Participant flow. De = demographics, T = transmission of the death news, P = psychopathology: major depression, bipolar disorder, manic episode, borderline personality disorder, posttraumatic disorder, alcohol or substance misuse, psychotic episode, D = depression, A = anxiety, Su = suicidality, C = cognitions; G = grief, St = stigmatization; SS = social support, E = Evaluation of the intervention

The flow of participants through the study is depicted in Fig. 1. Participants who are interested in the treatment can obtain general information about the program on the study website (www.hilfe-nach-suizid.de). This website provides extensive information on relevant topics for people bereaved by suicide. In addition, visitors to the website receive further information on helpful services (e.g. psychotherapy, counseling) and recommendations of useful literature.

To take part in the online intervention, participants first have to register on the website. Afterwards they will receive comprehensive information about the process of the study and a consent form for participation. As soon as participants have signed the consent form and have returned it (e.g. email, fax, or by post) they are invited to an online survey, which contains several questionnaires for diagnostic purposes (Table 1). To further examine the inclusion and exclusion criteria, participants will take part in a clinical telephone interview. This interview tests for acute suicidality, severe depression, acute psychotic experiences, bipolar disorder, PTSD, and BPD. Participants who do not meet the inclusion criteria receive further information on psychotherapy, counseling or self-help groups. In the case of clearly elevated scores on the measurements, participants are debriefed and are advised to see a doctor or psychotherapist. If the interviewer finds evidence of acute suicidality, he or she engages in crisis management strategies [35] or will arrange another telephone meeting to monitor the process. There are no specific provisions for the post-trial care of participants. If participants require further support after post-trial measurements, they are recommended by the project coordinators to seek further psychosocial support.

**Table 1:**
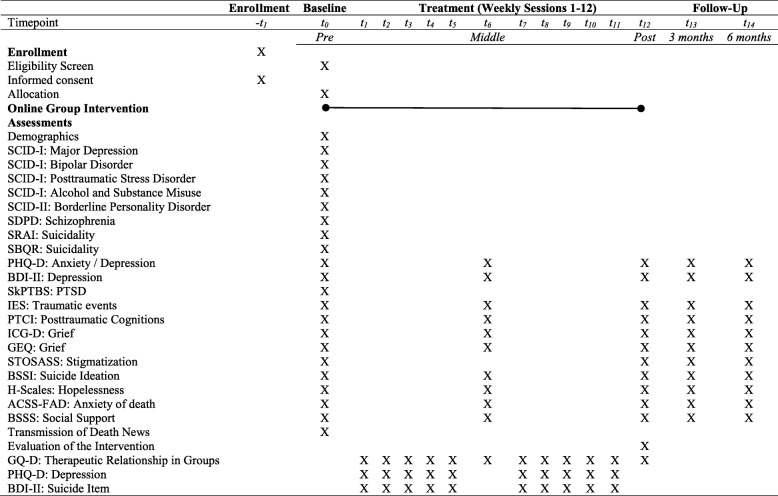
Schedule of enrollment, interventions, and assessments (SPIRIT Figure)

SCID-I = Structured Clinical Interview for DSM-IV Axis I; SCID-II = Structured Clinical Interview for DSM-IV Axis II; SDPD = Dutch Screening Device for Psychotic Disorder; SRAI = Suicide Risk Assessment Interview; SBQR = Suicide Behaviors Questionnaire Revised; PHQ-D = German Patient Health Questionnaire; BDI-II = Becks Depression Inventory-II; SkPTBS = Screening for complex PTSD; IES = Impact of Event Scale; PTCI = Posttraumatic Cognitions Inventory; ICG = Inventory of Complicated Grief; GEQ = Grief Experience Questionnaire; STOSASS = Stigma of Suicide and Suicide Survivor Scale; BSSI = Beck Scale for Suicide Ideation; H-Scales = H-Scales to Assess Hopelessness; ACSS-FAD = Revised Acquired Capability of Suicide Scale; BSSS = Berlin Social Support Scale; GQ-D = German Group Questionnaire

### Randomization

We will use block randomization without extra stratification: As soon as 10 participants are included, they are considered to form a group. A researcher (first author) who is not involved in participant recruitment, clinical assessments, or treatment, obtains the information by e-mail that a new group is complete. Then, the whole group is randomly assigned to either the treatment or the waitlist control group. The researcher then executes the randomization using a generated list at www.random.org. The person who is allocating the participants thus has no influence on the randomization and enrollment process. Following randomization, participants will be notified of their allocation.

### Blinding

Because of the high level of organizational effort involved in randomizing groups, it is not possible to blind the participants, group leaders, or study employees. The data analysis will be blinded. Thus, an independent researcher will conceal the participants’ IDs and their group assignment in the data set (i.e. relabel the variables). After all results are finalized, the independent researcher will reveal the correct group identification to the authors.

### Symptom assessment

During the intervention, participants are requested to complete online surveys to assess potential changes in the therapeutic process. More precisely, there are 15 measurement points (Table 1): at the initial stage (pre-assessment (T0)), after each group session (T1–T12 including a treatment mid-point assessment after 6 weeks (T6), and a post-assessment at the end of the intervention (T12)), after 3-month follow up (T13), and finally, after 6-month follow up (T14).

### The online group intervention

The online group intervention is manualized and consists of 12 weekly sessions each lasting 90 min. Each group has a maximum of 10 participants and is guided by a psychotherapist (or a psychotherapist in training) and a self-help counsellor who has herself or himself been bereaved by suicide. The participation is voluntary and free. Participants are allowed to skip sessions or to drop out of the program at any time.

### Development of the intervention

#### Focus groups

Three focus groups were conducted in order to develop the intervention. The composition of the groups was based on gender, age and kinship to the deceased. The minimum age was 18 years, and the time since the suicide had to be at least 1 year. The 23 recruited participants were on average 53 years old (SD 13, range 20–72) and 86% were women. Most participants had lost a child (52%), followed by a spouse or partner (24%), a parent or sibling (each 10%), or others (5%). Almost all participants had received support at the time of measurement, namely from psychotherapy (86%), self-help group (48%), a doctor (29%), pastoral care (19%), or grief counseling (14%).

The focus group participants were asked what they believed were difficulties during their grief process and what were effective strategies to find meaning in relation to the suicide. Further, they were interviewed as to which themes are relevant to be included in an Internet-based intervention. The group conversations lasted 4 h with a 1-h break and were recorded in audio format, transcribed, and evaluated with respect to important themes for a group intervention.

#### Testimonial videos

Most of the sessions start with a testimonial video in which a person bereaved by suicide talks about her or his own experiences of the session topic (e.g. guilt, own suicidality). The screenplays of the protagonist’s monologue (played by an actor/actress) were developed with excerpts of the transcriptions from the focus groups. In order to ensure a balanced gender ratio, both men and women were represented equally in the testimonial videos. All testimonial videos follow the same structure: (1) How did the bereaved person experience e.g. feelings of guilt in the first days and weeks after the loss? (2) How did the bereaved person experience feelings of guilt over the course of time? (3) How did the bereaved person experience feelings of guilt today? The protagonists in the testimonial videos aim to function as role models in dealing with difficult emotions that occur after a suicide.

#### Treatment modules

The selection of the relevant themes for the intervention were based on the literature on grief or treatment of traumatized people [34], and on the results of the focus groups. Focus group interviews were analyzed and relevant themes were included in the treatment manual. Table 2 describes the complete content by session. The manual is based on cognitive behavioral principles and consists of (1) testimonial videos, (2) psychoeducation (e.g. grief, guilt, suicidality), (3) a guided group discussion about the topic of the session, and (4) individual tasks between the sessions (e.g. writing a farewell letter to the deceased).

**Table 2:** The online group intervention: content and structure of each session

Number	Content	Structure
1	Introduction	▪ Group discussion: participants introduce themselves and the deceased ▪ Group guides introduce the program ▪ Individual writing assignment: set grief-related therapy goals
2	The suicide mode	▪ Video: a psychiatrist explains physical and psychological aspects of a suicide ▪ Group discussion on the suicide ▪ Individual writing assignment: circumstances of the suicide
3	My grief	▪ Psychoeducation: grief tasks [36] ▪ Group discussion on grief ▪ Individual writing assignment: relationship with the deceased person
4	Suicide as traumatic event	▪ Psychoeducation: symptoms of post-traumatic stress reactions ▪ Group discussion on symptoms ▪ Individual writing assignment: last hours of the deceased person
5	Meaning and the why-question	▪ Video: a wife talks about meaning-making after the suicide ▪ Group discussion on rumination ▪ Individual writing assignment: positive behavioral activation
6	Guilt	▪ Video: a mother talks about coping with guilt ▪ Group discussion on the functionality of guilt ▪ Individual writing assignment: letter to a friend
7	Own suicidality	▪ Video: a mother talks about coping with her own suicidality ▪ Psychoeducation: warning signs, coping strategies ▪ Group discussion on suicidality ▪ Individual writing assignment: crisis plan
8	Shame and stigmatization	▪ Video: a father talks about coping with stigmatization ▪ Group discussion on stigmatization ▪ Individual writing assignment: coping with stigmatizing reactions
9	To communicate the suicide	▪ Video: a brother talks about his way to communicate with others about the suicide of his sibling ▪ Psychoeducation: gender differences, communication styles ▪ Group discussion on communication ▪ Individual writing assignment: letter to a friend
10	Grief rituals	▪ Psychoeducation: grief rituals ▪ Group discussion on rituals ▪ Individual writing assignment: letter to the deceased person
11	Finding a new role in the future	▪ Video: a daughter talks about how she regained her life after the loss of her father ▪ Group discussion on coping with grief in the future and plans for the future ▪ Individual writing assignment: letter from the deceased person
12	Goodbye	▪ Group discussion about the intervention, changes and the group experience ▪ Individual writing assignment: goal attainment

#### The Internet tool

The webinar will be held using the Internet tool Adobe Connect®. Participants receive a link to log onto an online-seminar room and log in with a self-chosen user name. They are connected with each other via microphones and headphones but without video transmission. The visual transmission is disconnected to ensure increased anonymity for the participants. The participants are able to see PowerPoint slides or videos, which are part of the treatment program on their computer screen. The two group leaders can additionally access a list of names of all group members. There is also a chat option, which can be used by group participants or group leaders.

### Measures

#### Screening for eligibility criteria

The study coordinators will screen the participants for eligibility criteria using a semi-standardized clinical telephone interview. To exclude the presence of a current major depressive or manic episode, bipolar disorder, BPD, PTSD, or alcohol and substance misuse, the corresponding sections from the German version of the Structured Clinical Interview for DSM-IV [37] will be applied. The inter-rater reliabilities of the diagnoses vary between fair (e.g. agoraphobia) and excellent (e.g. PTSD, BPD) scores [38]. In addition, the German translations [39] of the Dutch Screening Device for Psychotic Disorder [40] and the Suicide Risk Assessment Interview [41] will be used to screen for acute schizophrenia and suicidality, respectively. All outcomes will be aggregated using sum scores, means, or standardized mean differences (i.e., Cohen’s *d* value for effect size) from baseline to post intervention and follow up. Thus, the timeframe will be 12 weeks from pre to post intervention, 3 months from pre to follow-up 1 and 3 months from pre to follow-up 2. The description of the specific outcome information (i.e. domain, measurement) can be found below.

#### Primary outcomes

The primary outcomes will be the level of suicidality and depression post treatment, and at 3-month and 6-month follow up. Suicidality and depression will be assessed using the following instruments.

##### Revised Acquired Capability of Suicide Scale (ACSS-FAD)

The ACSS-FAD [42] instrument assesses fearlessness of death, with seven items using a 5-point rating scale (1 = “does not apply at all to me”, 5 = “applies completely to me”). It had a good internal consistency (α = .79) in a German student sample.

##### Beck Depression Inventory (BDI-II)

The BDI-II [43] is one of the most used inventories to examine the severity of depression during the 2 weeks preceding the test. There are 21 symptom groups, and participants chose one statement out of four to seven response categories (coded 0, 1, 2, and 3) per symptom. Sum scores of 29 indicate severe depression. Internal consistencies were α = .90 in a healthy sample and α = .93 in a client sample, respectively.

##### Beck Scale for Suicide Ideation (BSS)

Suicidal thinking is assessed with the BSS [44, 45], which has 21 symptom groups, each with three different response categories, representing a 3-point rating scale. The first five groups are for screening purposes and the last two groups are not included in the total BSS sum score. Higher sum scores (range 0–38) indicate a higher risk of suicide. The internal consistency in a non-clinical German sample was very good (α = .94).

#### Secondary outcomes

As secondary outcomes, prolonged grief disorder, PTSD, hopelessness, anxiety, perceived stigmatization, and perceived social support will be assessed post treatment, at 3-month follow up, and 6-month follow up. These variables will be assessed using the following instruments:

##### Short version of the Patient Health Questionnaire (PHQ-D)

The short version of the PHQ-D [46] is a 15-item screening test for depressive disorders (nine items, 4-point rating scale: 1 = “not at all”, 4 = “almost every day”), panic disorders, (i.e. panic attacks, five items, dichotomous rating scale: 1 = “no”, 2 = “yes”) and psychosocial impairment (one item, 4-point rating scale: 1 = “not at all impaired”, 4 = “very impaired”). Graefe, Zipfel, Herzog, and Loewe [47] report a satisfactory Cronbach’s alpha of α = .88 for the depression scale of the PHQ-D in a German clinical sample.

##### Screening for Complex PTSD (SkPTBS)

The SkPTBS [48] is a screening instrument for complex PTSD, with 34 items including traumatic events (self or witness) and reactions to the most distressing event, on a 7-point rating scale (1 = “not at all”, 7 = “completely”). The internal consistency was α = .91 in a German clinical sample.

##### Revised Impact of Event Scale (IES-R)

We will assess posttraumatic stress reactions within the last 7 days using the IES-R [49], which consists of the three subscales *intrusion* (seven items), *avoidance* (eight items), and *hyperarousal* (seven items) and uses a 4-point rating scale (1 = “not at all”, 4 = “often”). Internal consistency for each subscale was reported for patient groups and vary between α = .71 and α = .90.

##### Posttraumatic Cognitions Inventory (PTCI) 50]

The German version of the PTCI [50] is a 33-item questionnaire that assesses dysfunctional cognitions of traumatic events on a 7-point rating scale (1 = “totally disagree”, 7 = “totally agree”). In a clinical sample, the three subscales - negative cognitions about the self (21 items), negative cognitions about the world (seven items), and self-blame (five items) – have been shown to have satisfactory to very good internal consistency: α = .97, α = .88, and α = .86, respectively.

##### Inventory of Complicated Grief (ICG-D)

The German version [51] of the ICG [52] measures the extent of complicated grief symptoms (synonyms: prolonged grief, persistent complex bereavement disorder [53];) with 19 items on a 5-point rating-scale (1 = “never”, 5 = “always”). Cronbach’s alpha was .87 in a clinical sample.

##### Grief Experience Questionnaire (GEQ)

The GEQ [54] is a questionnaire that measures reactions to grief and symptoms that are typical in suicide bereavement. In the current study, only the stigmatization subscale is used. This scale has 10 items on a 5-point rating scale (1 = “never”, 5 = “almost always”) with a good Cronbach’s alpha value (α = .86) in a sample of bereaved students. The original items were translated into German by the last author and another research assistant using back and forth translation (see Additional file 3).

##### Stigma of Suicide and Suicide Survivor (STOSASS)

To evaluate the level of perceived stigmatization towards completed suicide, we applied the STOSASS [55], which comprises the two subscales “stigma towards the suicidal person” and “stigma towards the suicide survivor”. Participants indicate their agreement with 17 items on a 4-point rating scale (1 = “strongly disagree”, 4 = “strongly agree”). Internal consistency for the subscales in non-clinical and clinical samples was good (between .79 to .83). The original items were translated into German by the last author and another research assistant using back and forth translation (see Additional file 4).

##### H-Scales

The H-Scales [56] are a German translation of the Beck Hopelessness Scale proposed by Beck, Weissman, Lester, and Trexler [57]. The short version with 10 items to measure the participants’ negative expectations of themselves, the environment and their future life on a six-point rating scale (1 = “completely wrong”, 6 = “completely correct”) will be used. Cronbach’s alpha ranged between α = .74 and α = .92 in clinical and non-clinical samples.

##### Berlin Social Support Scales (BSSS)

The BSSS [58] assesses social support using five subscales: perceived available social support (eight items, α = .83), actually received social support (eleven items, α = .83), need for support (four items, α = .63), mobilization of social support (five items, α = .819, and protective buffering (six items, α = .82). The questionnaire was validated in a clinical sample and uses a 4-point rating scale (1 = “is not correct”, 4 = “is completely correct”). In the current study, only the subscales for perceived available social support, need for support, and mobilization of social support will be used.

##### Questionnaire on Police Delivery of Death News (QPDDN)

The QPDDN (Hofmann and Wagner) is a newly constructed questionnaire to examine how mourners perceive the behavior of police officers who transmit the news of the death. The questionnaire consists of 34 items with items of perceived stigmatization, experienced emotional support, and perceived behavior of the police (see Additional file 5) with two items using a 6-point rating (1 = “not correct at all”, 6 = “completely correct”). Seven items assess additional information on information material received, how the message was delivered, if there was any support (e.g. doctor, pastor), which behavior they found most disturbing and what they would have wished for. Information on the validity and reliability will be provided once the data have been analyzed.

#### Process measures

In addition to the primary and secondary outcomes, we are also interested in variables that relate to the therapeutic process and the development of suicidal or depressive markers from one treatment session to another. We expect that the depressive and suicidal symptoms will reduce over the course of the intervention. After each session, depression will be assessed using the depression subscale from the PHQ-D [46]. Suicidality will be measured using the “suicide item” from the BDI-II [43].

Furthermore, the therapeutic relationships within the intervention groups are assessed using the German version of the Group Questionnaire (GQ-D) [59]. This inventory consists of subscales for positive bonding relationship (α = .92), positive working relationship (α = .89), and negative relationship (α = .79) and differentiates between group members, group leaders, and the group as a whole. Agreement with the statements is indicated on a 7-point rating scale (1 = “is not correct at all”, 7 = “is completely correct”).

### Data management and storage

#### Personal data

As part of the registration process, participants provide their name, telephone number, email address, and home address. This information is pseudonymized using encrypted codes. The pseudo-anonymization will be saved in a coding list, recorded on paper and only in one version. This list will be locked up with no third-party access. As long as the coding list exists, the study participants are able to request the deletion of all data collected from them. The coding list will be destroyed after data collection. Then all data from the study will be completely anonymous and the personal data of participants can no longer be identified.

Furthermore, we assess sociodemographic data, symptoms of general psychopathology, grief, or suicidality within the telephone interview and the online surveys. We conduct all surveys using the program Unipark®. The study-related data are stored and evaluated according to legal provisions, without the participants name or IP address. The data will be transferred to the statistical program SPSS 20. We keep the data in a place to which only employees of this study have access. We store the digital data on secured servers at the Medical School Berlin for at least 10 years. We will delete the data once the retention obligation has expired.

The audio recordings that were made in the context of the focus groups will be deleted after the transcription has been completed. The transcription has been anonymized. There will be no biological specimens collected at any time.

### Statistical analysis

We will use repeated measures analysis of variance (ANOVA) to analyze the changes in the primary endpoint variables (suicidality and depression) and the secondary outcomes from baseline to post-measurement and the two follow-up assessments (3 months, 6 months), where the dependent variable will be the treatment condition (treatment group vs. waitlist control group). In addition, we will control for the baseline levels of depression and suicidality using analysis of covariance (ANCOVA). The predictor analyses will be conducted using the method of multiple regression. Interim analyses will be calculated to present first results before the study is finished. To control for differences between the online groups, multilevel analyses will be conducted to allow for variation between participants and online groups at level 1. The assignment to the study group (i.e., treatment, wait list) will be on level 2. Intraclass correlation will be tested to examine potential clustering effects of the online groups.

### Handling of missing data

We will follow the principles of intention-to-treat analysis. Participants who drop out of the study after randomization are asked to state their reasons for doing so and are asked to continue the assessments. Missing values will be imputed using the approach of last observation carried forward. In the case of a participant dropping out during the intervention, the participant will be asked to fill in the post-questionnaire, and the reason for dropping out will be assessed.

### Monitoring, ethical considerations, and safety

The group leaders record each session so that the study employees can randomly examine the adherence to the intervention manual. In addition, the group leaders document each session (e.g. attendance, mood of participants, disturbances) and contact participants individually via the study online portal in the case of absence, problematic behavior, suicidality, or deterioration of symptoms. In addition, the study employees receive an automatic email when participants report increased levels of suicidality in the questionnaires after each session. If suicidality or adverse effects are suspected, the study employees will call the participants once a week to monitor and assess their mood and offer support.

The randomized controlled trial was approved by the ethics committee of Medical School Hamburg (03.03.2018). Participants are informed in writing (i.e. study information, consent form) about the procedure of the program, the aims of the study, the inclusion and exclusion criteria, risks and costs of participation, confidentiality, and data storage. They have the opportunity to drop out of the program at any time without giving reasons. They are also allowed to use other assistance (e.g. psychotherapy) in addition to study participation.

The participants are also informed about the possibility of detecting abnormalities in the clinical interview (before treatment) or during the data analysis. In that case, we contact participants for a more detailed clinical assessment. If we find clinically relevant aspects or diagnoses, the participants will be informed. However, we point out that study assessments do not substitute clinical diagnosis by a medical doctor or psychotherapist.

During treatment, participants may experience a worsening in mood and sadness at first when they deal with the suicide of their loved ones. Such mood aggravations are normal in an intensive grief process and usually exist only for a short time. In the case of acute suicidality (i.e. corresponding hints in the suicidality item of the BDI-II), we contact the participant by telephone and offer counseling. He or she may be excluded from the program and referred to local professionals. However, the scientific evidence so far suggests that the proposed online program can reduce difficulties in coping with grief reactions and symptoms. Hence, we do not expect any unwanted side effects or lasting psychopathological worsening at the end of the program. Additionally, a supervisor will provide clinical supervision every fourth session to ensure treatment adherence and provide clinical support.

There will be no external data monitoring committee because the Sponsor and cooperation partners will function whereby procedures, results, and modifications have to be communicated on a regular basis (at least twice annually). Although the execution of the study and the data analyses are independent of these institutions, we will write several interim reports about the achievement of project aims, results, and finances and justify deviations. If adverse events arise in the suggested procedures (e.g. eligibility criteria, randomization strategy, parts of the intervention, etc.) the study coordinators will discuss the necessity and impact of possible changes to the study protocol. An audit is not scheduled in our study.

## Discussion

The primary aim of the study is to reduce suicidality and depression in people bereaved by suicide, using an online-group intervention. Furthermore, the intervention aims to reduce secondary parameters such as prolonged grief, anxiety, or PTSD. Last but not least, we are also interested in sociodemographic predictors (e.g. age, sex, relationship to the deceased) that predict successful treatment and in the cognitive processes (e.g. experience of stigmatization, guilt) that influence the participants’ general psychopathology.

People bereaved by suicide have a high risk of developing mental illnesses and committing suicide themselves [10]. For this reason, it is important to provide adequate low-threshold support. One of the main advantages of the program is that it is easy to access and geographically independent. The attendance of the participants and the group leaders is possible without much technical knowledge and without any special equipment. Furthermore, participants can largely remain anonymous, which might decrease hesitation to seek professional help, as after suicide people often face stigmatization in their environment. The advantages mentioned above also apply to the group leaders. Further, the intervention offers the unique opportunity to meet other people after suicide bereavement, which reduces social isolation.

With a planned sample size of more than 70 participants, this trial is adequately powered for detecting a medium effect size for the comparison between the treatment and the waitlist group. Additionally, we explore multiple predictors of treatment-outcome moderator variables to define detailed processes of the effectiveness.

The study has multiple methodological strengths such as block randomization and the use of a standardized manual with precise procedures and instructions that are based on cognitive behavioral principles. The group leaders receive detailed training and regular supervision to ensure treatment fidelity. Because the intervention is led by a psychologist and a self-help counselor who has also experienced suicide loss, both perspectives are considered, thus complementing each other’s work. Last but not least, this study uses validated questionnaires and an additional clinical interview in the diagnostic process to make sure the participants meet the inclusion criteria.

Despite its strength, some limitations of the current study design are worth mentioning. First, the study does not test whether the online group intervention is more effective than standard psychotherapy or self-help groups, because we use a waitlist control group design. Future research should expand this approach to active treatment conditions. Second, the organization of groups is not always without difficulties (e.g. temporary delays, schedule difficulties). This could mean that participants have to be randomized a second time if they cannot take part in the assigned group appointment. Further, participants may seek professional support in addition to our program. This might affect the effect sizes of our intervention although we control for such influences (e.g. record additional psychotherapy, medication, self-help groups). Follow-up studies or dismantling studies might further investigate which aspects of the intervention in particular drive the effects.

Taken together, this study and its results will help to better understand the problems faced by people bereaved by suicide. We expect our online group intervention to be effective in reducing parameters like depression, suicidality, and prolonged grief disorder. Hopefully, the knowledge thus gained can be used for further studies in this target group.

## Dissemination

We will present the results of the study to the scientific community in peer-reviewed journals and at international and national conferences. We will report the results to the non-scientific community by publishing them on our website, in newspaper articles, in newsletters for VEID and AGUS, and in magazines for practitioners. Participants interested in the study results will receive a report via email.

## Trial status (version 1 19/08/2019)

We started recruitment in October 2018.

Recruitment will be finished approximately by May 2020.

## Supplementary information

**Additional file 1.** SPIRIT Checklist (2013): Recommended items to address in a clinical trial protocol and related documents. (DOCX 45 kb)

**Additional file 2.** Informed Consent Form (Sample) and Study Information (Sample)

**Additional file 3.** German Translation of the Grief Experience Questionnaire.

**Additional file 4.** German Translation of the Stigma of Suicide Attempt and the Stigma of Suicide and Suicide Survivor Scales.

**Additional file 5. ** Questionnaire on Police Delivery of Death News.

## Data Availability

The datasets generated and/or analyzed during the current study are available from the corresponding author on reasonable request.

## Abbreviations

ACSS-FAD: Revised Acquired Capability of Suicide Scale
AGUS: Angehörige um Suizid e.V.
BDI-II: Beck Depression Inventory-II
BPD: Borderline personality disorder
BSS: Beck Scale for Suicide Ideation
BSSS: Berlin Social Support Scales
GEQ: Grief Experience Questionnaire
GQ: Group Questionnaire
H-Scales: Hopelessness Scale
ICG-D: Inventory of Complicated Grief-German Version
IES-R: Revised Impact of Event Scale
PHQ-D: Short Version of the Patient Health Questionnaire-German Version
PTCI: Posttraumatic Cognitions Inventory
PTSD: Posttraumatic stress disorder
QPDDN: Questionnaire on Police Delivery of Death News
SkPTBS: Screening for complex posttraumatic stress disorder
SPIRIT: Standard Protocol Items: Recommendations for Interventional Trials
STOSASS: Stigma of Suicide and Suicide Survivor
VEID: Bundesverband Verwaiste Eltern und trauernde Geschwister e.V.
