# Supplementary material for: Online-group intervention after suicide bereavement through the use of webinars: study protocol for a randomized controlled trial
Source: Trials. 2020 Jan 8;21:45. doi: 10.1186/s13063-019-3891-5 (PMC6951011; doi:10.1186/s13063-019-3891-5)
Supplement: Supplementary file 2 — Additional file 2. Informed Consent Form (Sample) and Study Information (Sample) [file 13063_2019_3891_MOESM2_ESM.docx]

Appendix B

Informed Consent Form (Sample) and Study Information (Sample)
