# Supplementary material for: Online-group intervention after suicide bereavement through the use of webinars: study protocol for a randomized controlled trial
Source: Trials. 2020 Jan 8;21:45. doi: 10.1186/s13063-019-3891-5 (PMC6951011; doi:10.1186/s13063-019-3891-5)
Supplement: Supplementary file 3 — Additional file 3. German Translation of the Grief Experience Questionnaire. [file 13063_2019_3891_MOESM3_ESM.docx]

Appendix C

German Translation of the Grief Experience Questionnaire

**Wie oft haben Sie seit dem Suizid…**

*Rating Skala: 1 = Nie, 2 = Kaum, 3 = Manchmal, 4 = Oft, 5 = Fast immer*

| **No** | **Originalitem** | **Deutsche Übersetzung**  **(Katja Mériau)** | **Englische Rückübersetzung (Ulrike Maaß)** | **Revision der deutschen Items nach englischer Rückübersetzung** | **Subskala** | **No** |  |  |
| --- | --- | --- | --- | --- | --- | --- | --- | --- |
| 1 | Think that you should go see a doctor.** | Denken, dass Sie einen Arzt aufsuchen sollte. | Think that you should see a doctor. | gedacht, dass Sie einen Arzt aufsuchen sollten. | Somatische Reaktionen | 1 |  |  |
| 2 | Experience feeling sick. | Sich schlecht fühlen. | feel bad. | sich krank gefühlt. | Somatische Reaktionen | 2 |  |  |
| 3 | Experience trembling, shaking, or twitching. | Sich zittrig, zitternd oder zuckend fühlen. | feel shaky, trembling, or twitchy | sich zittrig, zitternd oder zuckend gefühlt. | Somatische Reaktionen | 3 |  |  |
| 4 | Experience light-headedness, dizziness, or fainting. | Sich benommen, schwindelig oder einer Ohnmacht nahe fühlen. | feel numb, dizzy, or feel faint | sich benommen, schwindelig oder einer Ohnmacht nahe gefühlt. | Somatische Reaktionen | 4 |  |  |
| 5 | Experience nervousness. | Sich nervös fühlen. | feel nervous | sich nervös gefühlt. | Somatische Reaktionen | 5 |  |  |
| 6 | Think that people were uncomfortable offering their condolences to you. | Denken, dass es anderen unangenehm war, Ihnen ihr Beileid auszusprechen. | Think that others felt uncomfortable to offer their condolences | gedacht, dass es anderen unangenehm war, Ihnen ihr Beileid auszusprechen. | Stigmatisierung | 6 |  |  |
| 7 | Avoid talking about the negative or unpleasant parts of your relationship. | Vermeiden, über die negativen oder unerfreulichen Aspekte Ihrer Beziehung zur verstorbenen Person zu sprechen. | Avoid talking about the negative or unpleasant aspects of your relationship with the deceased person | vermieden, über die negativen oder unerfreulichen Aspekte Ihrer Beziehung zu sprechen. | Scham | 7 |  |  |
| 8 | Feel like you just could not make it through another day. | Sich fühlen, als ob Sie keinen weiteren Tag überstehen könnten. | Feel as if you could not survive [get through] another day. | sich gefühlt, als ob Sie keinen weiteren Tag überstehen könnten. | Selbstzerstörerische Orientierung | 8 |  |  |
| 9 | Feel like you would never be able to get over the death. | Sich fühlen, als ob Sie niemals über den Tod der Person hinwegkämen. | Feel as if you could never get over the persons death | sich gefühlt, als ob Sie niemals über den Tod der Person hinwegkämen. | Suche nach Erklärungen | 9 |  |  |
| 10 | Feel anger or resentment toward the person after the death. | Wut oder Verbitterung der verstorbenen Person gegenüber fühlen. | Feel anger and resentment [bitterness] against the deceased person | Wut oder Verbitterung der verstorbenen Person gegenüber gefühlt. | Aufgabe/Ablehnung | 10 |  |  |
| 11 | Question why the person had to die. | Sich fragen, warum die Person sterben musste. | Ask yourself why the person had to die | Sich gefragt, warum die Person sterben musste. | Suche nach Erklärungen | 11 |  |  |
| 12 | Find you couldn’t stop thinking about how the death occurred. | Feststellen, dass Sie nicht aufhören konnten nachzudenken, wie der Tod passierte. | Realize that you could not stop thinking about how the death occurred | bemerkt, dass Sie nicht aufhören konnten nachzudenken, wie der Tod passierte. | Suche nach Erklärungen | 12 |  |  |
| 13 | Think that the person’s time to die had not yet come. | Denken, dass es für die Person noch nicht Zeit war zu sterben. | Think that it was not time for the person to die | gedacht, dass die Zeit für die Person noch nicht gekommen war, zu sterben | Suche nach Erklärungen | 13 |  |  |
| 14 | Find yourself not accepting the fact that the death happened. | Erkennen, dass Sie nicht akzeptieren können, dass der Tod tatsächlich geschah. | Realize that you cannot accept that the death actually happened | bemerkt, dass Sie nicht akzeptieren können, dass der Tod tatsächlich geschah. | Suche nach Erklärungen | 14 |  |  |
| 15 | Try to find a good reason for the death. | Versuchen, einen guten Grund für den Tod zu finden. | Trie to find a good reason for the death | versucht, einen guten Grund für den Tod zu finden. | Suche nach Erklärungen | 15 |  |  |
| 16 | Feel avoided by friends. | Sich von Freunden gemieden fühlen. | Feel avoided by friends | sich von Freunden gemieden gefühlt. | Stigmatisierung | 16 |  |  |
| 17 | Think that others didn’t want you to talk about the death. | Denken, dass andere nicht wollten, dass Sie über den Tod redeten. | Think that others don’t want you to talk about the death | gedacht, dass andere nicht wollten, dass Sie über den Tod reden. | Stigmatisierung | 17 |  |  |
| 18 | Feel like no one cared to listen to you. | Sich fühlen, als ob niemand sich bemühte Ihnen zuzuhören. | Feel as if nobody cared listening [made an effort to listen] | sich gefühlt, als ob niemand sich bemühte, Ihnen zuzuhören. | Stigmatisierung | 18 |  |  |
| 19 | Feel that neighbors and in-laws did not offer enough concern. | Sich fühlen, als ob Nachbarn oder angeheiratete Verwandte sich nicht besorgt genug zeigten. | Feel as if neighbors or in-laws did not show themselves to be worried enough | sich gefühlt, als ob Nachbarn oder angeheiratete Verwandte nicht genug Anteilnahme zeigten. | Stigmatisierung | 19 |  |  |
| 20 | Feel like a social outcast. | Sich wie ein Außenseiter fühlen. | Feel like an outsider | sich wie ein Außenseiter der Gesellschaft gefühlt. | Stigmatisierung | 20 |  |  |
| 21 | Think people were gossiping about you or the person. | Denken, dass andere über Sie oder die verstorbene Person tratschen. | Think that others gossip about you or the deceased person | gedacht, dass andere über Sie oder die verstorbene Person tratschen. | Stigmatisierung | 21 |  |  |
| 22 | Feel like people were probably wondering about what kind of personal problems you and the person had experienced. | Sich fühlen, als ob andere sich womöglich fragten, welche Art von Problemen Sie und die verstorbene Person hatten. | Feel as if others may have been wondering what kind of problems you and the deceased person had | sich gefühlt, als ob andere sich womöglich fragten, welche Art von persönlichen Problemen Sie und die verstorbene Person hatten. | Stigmatisierung | 22 |  |  |
| 23 | Feel like others may have blamed you for the death. | Sich fühlen, als ob andere Sie für den Tod der Person verantwortlich machten. | Feel as if others blame you for the persons death | sich gefühlt, als ob andere Sie für den Tod der Person verantwortlich machten. | Verantwortung | 23 |  |  |
| 24 | Feel like the death somehow reflected negatively on you or your family.* | Sich fühlen, als ob der Suizid ein schlechtes Licht auf Sie oder Ihre Familie geworfen hätte. | Feel as if the suicide had put you or your family in a bad light | sich fühlt, als ob der Suizid ein schlechtes Licht auf Sie oder Ihre Familie geworfen hätte. | Aufgabe/Ablehnung - Stigmatisierung | 24 |  |  |
| 25 | Feel somehow stigmatized by the death. | Sich durch den Suizid in irgendeiner Form stigmatisiert fühlen. | Feel stigmatized by the suicide in any way | sich durch den Suizid in irgendeiner Form stigmatisiert gefühlt. | Stigmatisierung | 25 |  |  |
| 26 | Think of times before the death when you could have made the person’s life more pleasant. | An Zeiten vor dem Suizid denken, als Sie der verstorbenen Person das Leben noch angenehmer hätten machen können. | Think about times before the suicide when you could have make life more pleasant for the deceased person | an Zeiten vor dem Suizid gedacht, als Sie der verstorbenen Person das Leben noch angenehmer hätten machen können. | Schuld | 26 |  |  |
| 27 | Wished that you hadn’t said or done certain things during your relationship with the person. | Sich wünschen, man hätte bestimmte Dinge während der Beziehung mit der verstorbenen Person nicht gesagt oder getan. | Wish that you hadn’t said or done certain things during the relationship with the deceased person | sich gewünscht, Sie hätten bestimmte Dinge während der Beziehung mit der verstorbenen Person nicht gesagt oder getan. | Schuld | 27 |  |  |
| 28 | Feel like there was something very important you wanted to make up to the person. | Sich fühlen, als ob Sie etwas sehr wichtiges bei der verstorbenen Person wieder gutmachen wollten. | Feel as if you wanted to make up for something important to the deceased person | sich gefühlt, als ob Sie etwas sehr wichtiges bei der verstorbenen Person wieder gutmachen wollten. | Schuld | 28 |  |  |
| 29 | Feel like maybe you didn’t care enough about the person. | Sich fühlen, als ob man sich vielleicht nicht genug um die verstorbene Person gekümmert hätte. | Feel as if you had not been taking care enough about the deceased person | sich gefühlt, als ob Sie sich vielleicht nicht genügend Gedanken um die verstorbene Person gemacht hätten. | Schuld | 29 |  |  |
| 30 | Feel somehow guilty after the death of the person. | Sich auf irgendeine Art schuldig fühlen seit dem Tod der Person. | Feel guilty in any way since the persons death | sich auf irgendeine Art schuldig gefühlt. | Schuld | 30 |  |  |
| 31 | Feel like the person had some kind of complaint against you at the time of the death. | Sich fühlen, als ob die Person zum Zeitpunkt ihres Todes irgendeine Art Klage gegen Sie vorzubringen hatte. | Feel as if the person had complaints against you at the time of her/his death | sich gefühlt, als ob die Person zum Zeitpunkt ihres Todes irgendeine Art Klage gegen Sie vorzubringen hatte. | Verantwortung | 31 |  |  |
| 32 | Feel that, had you somehow been a different person, the person would not have died. | Sich fühlen, als ob die Person noch leben würde, wenn Sie ein anderer Mensch gewesen wären. | Feel as if the person would still live if you had been a different person | sich gefühlt, als ob die Person sich nicht das Leben genommen hätte, wenn Sie ein anderer Mensch gewesen wären. | Verantwortung | 32 |  |  |
| 33 | Feel like you had made the person unhappy long before the death.* | Sich fühlen, als ob man die Person lange vor ihrem Tod unglücklich gemacht hätte. | Feel as if the you were making the person unhappy long before her/his death | sich gefühlt, als ob Sie die Person lange vor ihrem Tod unglücklich gemacht hätten. | Schuld / Verantwortung | 33 |  |  |
| 34 | Feel like you missed an early sign that may have indicated to you that the person was not going to be alive much longer. | Sich fühlen, als ob Sie frühe Anzeichen übersehen haben, die Ihnen vielleicht angezeigt hätten, dass die verstorbene Person nicht mehr lange leben würde. | Feel as if you missed early signs that may have indicated that the deceased person wouldn’t live much longer | sich gefühlt, als ob Sie frühe Anzeichen übersehen haben, die Ihnen vielleicht angezeigt hätten, dass die verstorbene Person nicht mehr lange leben würde. | Aufgabe / Ablehnung | 34 |  |  |
| 35 | Feel like the problems you and the person had together contributed to an untimely death. | Sich fühlen, als ob die Probleme, die Sie und die verstorbene Person hatten, zu ihrem verfrühtem Tod beigetragen hätten. | Feel as if the problems you and the deceased person had contributed to her/his early [premature] death | sich gefühlt, als ob die Probleme, die Sie und die verstorbene Person hatten, zu ihrem frühen Tod beigetragen hätten. | Verantwortung | 35 |  |  |
| 36 | Avoid talking about the death of the person. | Vermeiden über den Tod der Person zu sprechen. | Avoid talking about the persons death | vermieden über den Tod der Person zu sprechen. | Scham | 36 |  |  |
| 37 | Feel uncomfortable revealing the cause of the death. | Sich unwohl fühlen, die Todesursache zu offenbaren. | Feel uncomfortable revealing [disclosing] the cause of death | sich unwohl gefühlt, die Todesursache zu offenbaren. | Scham | 37 |  |  |
| 38 | Feel embarrassed about the death. | Sich für den Suizid schämen. | Being ashamed of the suicide | sich für den Suizid geschämt. | Scham | 38 |  |  |
| 39 | Feel uncomfortable about meeting someone who knew you and the deceased.** | Sich unwohl fühlen, jemanden zu treffen, der Sie und die verstorbene Person kannte. | Feel uncomfortable meeting someone who knew you and the deceased person | sich unwohl gefühlt, jemanden zu treffen, der Sie und die verstorbene Person kannte. | Scham | 39 |  |  |
| 40 | Not mention the death to people you met causally. | Den Tod flüchtigen Bekannten gegenüber zu verschweigen. | conceal the death from acquaintances | den Tod flüchtigen Bekannten gegenüber nicht erwähnt. | Scham | 40 |  |  |
| 41 | Feel like the person chose to leave you. | Sich fühlen, als ob die verstorbene Person sich entschieden hätte, Sie zu verlassen. | Feel as if the deceased person had decided to leave you | sich gefühlt, als ob die verstorbene Person es vorgezogen hat, Sie zu verlassen. | Aufgabe / Ablehnung | 41 |  |  |
| 42 | Feel deserted by the person. | Sich von der verstorbenen Person im Stich gelassen fühlen. | feel abandoned by the deceased person | sich von der verstorbenen Person im Stich gelassen gefühlt. | Aufgabe / Ablehnung | 42 |  |  |
| 43 | Feel that the death was somehow a deliberate abandonment of you. | Sich fühlen, als ob der Suizid ein vorsätzlicher Akt des Sie Verlassens sei. | Feel as if the suicide was a deliberate [intentional] act of leaving you [abondonment] | sich gefühlt, als ob der Suizid eine vorsätzliche Tat war, mit dem Ziel, Sie zu verlassen. | Aufgabe / Ablehnung | 43 |  |  |
| 44 | Feel that the person never considered what the death might do to you. | Sich fühlen, als ob die verstorbene Person nie bedacht hätte, was der Suizid Ihnen antun könnte. | Feel as if the deceased person had never thought about what the suicide might do to you | sich gefühlt, als ob die verstorbene Person nie bedacht hatte, was der Suizid Ihnen antun könnte. | Aufgabe / Ablehnung | 44 |  |  |
| 45 | Sense some feeling that the person had rejected you by dying. | Sich fühlen, als ob die verstorbene Person Sie durch ihren Tod zurückgewiesen hätte. | Feel as if the deceased person had rejected you with his/her death | sich gefühlt, als ob die verstorbene Person Sie durch ihren Tod zurückgewiesen hätte. | Aufgabe / Ablehnung | 45 |  |  |
| 46 | Feel like you just didn’t care enough to take better care of yourself. | Sich fühlen, als ob es Ihnen einfach nicht wichtig genug sei, sich besser um Sie selber zu kümmern. | Feel as if it was not important enough to take better care of yourself | sich gefühlt, als ob es Ihnen einfach nicht wichtig genug war, sich besser um Sie selber zu kümmern. | Selbstzerstörerische Orientierung | 46 |  |  |
| 47 | Find yourself totally preoccupied while you were driving.** | Feststellen, dass Sie während des Autofahrens völlig geistesabwesend sind. | Realize that you were completely absent-minded are during driving | bemerkt, dass Sie während des Autofahrens völlig geistesabwesend waren. | Selbstzerstörerische Orientierung | 47 |  |  |
| 48 | Worry that you might harm yourself. | Besorgt sein, dass Sie sich selber etwas antun würden. | Be worried that you might harm yourself [would do something to yourself] | sich gesorgt, dass Sie sich selber etwas antun würden. | Selbstzerstörerische Orientierung | 48 |  |  |
| 49 | Think of ending your own life. | Darüber nachdenken, Ihr eigenes Leben zu beenden. | Think about ending your own life | darüber nachgedacht, Ihr eigenes Leben zu beenden. | Selbstzerstörerische Orientierung | 49 |  |  |
| 50 | Intentionally try to hurt yourself. | Absichtlich versucht haben sich zu verletzen. | Intentionally tried to hurt yourself | absichtlich versucht haben, sich zu verletzen. | Selbstzerstörerische Orientierung | 50 |  |  |
| 51 | Wonder about the person’s motivation for not living longer. | Sich fragen, warum die verstorbene Person nicht mehr leben wollte. | Wonder why the deceased person did not want to live anymore | sich gefragt, welche Motivation die verstorbene Person hatte, nicht länger leben zu wollen | Aufgabe / Ablehnung | 51 |  |  |
| 52 | Feel like the person was somehow getting even with you by dying. | Sich fühlen, als ob Ihnen die verstorbene Person durch ihren Tod igrendetwas heimzahlte. | Feel as if the deceased person had paid you back for something | sich gefühlt, als ob Ihnen die verstorbene Person durch ihren Tod irgendetwas heimzahlte. | Aufgabe / Ablehnung | 52 |  |  |
| 53 | Feel that you should have somehow prevented the death. | Sich fühlen, als ob Sie irgendwie den Tod hätten verhindern sollen. | Feel as if you should have prevented the death somehow | das Gefühl, dass Sie irgendwie den Tod hätten verhindern müssen. | Aufgabe / Ablehnung | 53 |  |  |
| 54 | Tell someone that the cause of death was something different than what it really was. | Jemanden andere Todesumstände als die tatsächlichen mitteilen. | Communicate other circumstances of the death than the actual ones | jemanden eine andere Todesursache als die tatsächliche mitgeteilt. | Scham | 54 |  |  |
| 55 | Feel that the death was a senseless and wasteful loss of life. | Sich fühlen, als ob der Tod ein sinnloser Verlust und Vergeudung von Leben war. | Feel as if the death was a useless [meaningless] loss and a waste of life | sich gefühlt, als ob der Tod ein sinnloser und unnötiger Verlust von Leben war. | Suche nach Erklärungen | 55 |  |  |
| * Items assigned to two factors.  ** Items recommended for exclusion when scoring according to these dimensions. | | | | | | | | |

**Itemzuordnung zu den Subskalen**

| **Aufgabe / Ablehnung** | **Schuld** | **Verantwortung** | **Suche nach Erklärungen** | **Selbstzerstörerische Orientierung** | **Scham** | **Somatische Reaktionen** | **Stigmatisierung** |
| --- | --- | --- | --- | --- | --- | --- | --- |
| 10 | 26 | 23 | 9 | 8 | 7 | 1 | 6 |
| 24 | 27 | 31 | 11 | 46 | 36 | 2 | 16 |
| 34 | 28 | 32 | 12 | 47 | 37 | 3 | 17 |
| 41 | 29 | 33 | 13 | 48 | 38 | 4 | 18 |
| 42 | 30 | 35 | 14 | 49 | 39 | 5 | 19 |
| 43 | 33 |  | 15 | 50 | 40 |  | 20 |
| 44 |  |  | 55 |  | 54 |  | 21 |
| 45 |  |  |  |  |  |  | 22 |
| 51 |  |  |  |  |  |  | 24 |
| 52 |  |  |  |  |  |  | 25 |
| 53 |  |  |  |  |  |  |  |
