# Supplementary material for: Online-group intervention after suicide bereavement through the use of webinars: study protocol for a randomized controlled trial
Source: Trials. 2020 Jan 8;21:45. doi: 10.1186/s13063-019-3891-5 (PMC6951011; doi:10.1186/s13063-019-3891-5)
Supplement: Supplementary file 4 — Additional file 4. German Translation of the Stigma of Suicide Attempt and the Stigma of Suicide and Suicide Survivor Scales. [file 13063_2019_3891_MOESM4_ESM.docx]

Appendix D

German Translation of the Stigma of Suicide Attempt and the Stigma of Suicide and Suicide Survivor Scales

**Reference:**

Scocco, P., Castriotta, C., Toffol, E., & Preti, A. (2012). Stigma of Suicide Attempt (STOSA) scale and Stigma of Suicide and Suicide Survivor (STOSASS) scale: two new assessment tools. *Psychiatry Research, 200*(2), 872-878.

**STOSA**

| **Nr** | **Originalitem** | **Deutsche Übersetzung (Ulrike Maaß)** | **Englische Rückübersetzung (Katja Mériau)** | **Revision der deutschen Items nach englischer Rückübersetzung** |
| --- | --- | --- | --- | --- |
| 1 | Most people would willingly accept a person who attempted suicide as a close friend. | Die meisten Menschen würden bereitwillig eine Person, die einen Suizidversuch unternahm, als einen engen Freund annehmen. | Most people would readily accept a person who attempted suicide as a close friend. | Die meisten Menschen würden bereitwillig eine Person, die einen Suizidversuch unternahm, als enge_n Freund_in akzeptieren. |
| 2 | Most people believe that a person who attempted suicide is just as intelligent as the average person. | Die meisten Menschen glauben, dass eine Person, die einen Suizidversuch unternahm, genauso intelligent ist wie der Durchschnittsmensch. | Most people think that a person, who attempted suicide, is as intelligent as the average person. | Die meisten Menschen glauben, dass eine Person, die einen Suizidversuch unternahm, genauso intelligent ist wie der Durchschnittsmensch. |
| 3 | Most people believe that a person who attempted suicide is just as trustworthy as the average person. | Die meisten Menschen glauben, dass eine Person, die einen Suizidversuch unternahm, genauso vertrauenswürdig ist wie der Durchschnittsmensch. | Most people think that a person, who attempted suicide, is as trustworthy as the average person | Die meisten Menschen glauben, dass eine Person, die einen Suizidversuch unternahm, genauso vertrauenswürdig ist wie der Durchschnittsmensch. |
| 4 | Most people would accept a person who attempted suicide, and is healthy at the time, as a teacher of young children in a public school. | Die meisten Menschen würden eine Person, die einen Suizidversuch unternahm und derzeit gesund ist, als Lehrer an einer Schule für jüngere Kinder akzeptieren. | Most people would accept a person, who attempted suicide and is currently remitted, as a school teacher for younger children. | Die meisten Menschen würden eine Person, die einen Suizidversuch unternahm und derzeit gesund ist, als Grundschullehrer_in akzeptieren. |
| 5 | Most people feel that attempted suicide is a sign of personal failure. | Die meisten Menschen finden, dass ein Suizidversuch ein Zeichen für persönliches Versagen ist. | Most people think that a suicide attempt is a sign of personal failure. | Die meisten Menschen finden, dass ein Suizidversuch ein Zeichen für persönliches Versagen ist. |
| 6 | Most people would not hire a person who has attempted suicide to take care of their children, even if he or she has been well for some time. | Die meisten Menschen würden keine Person, die einen Suizidversuch unternahm, für die Versorgung ihrer Kinder einstellen – auch nicht, wenn es dieser Person seit einiger Zeit gut ginge. | Most people wouldn't hire a person who attempted suicide as a caretaker for their children, not even, if she has been well for some time. | Die meisten Menschen würden eine Person, die einen Suizidversuch unternahm, nicht für die Versorgung ihrer Kinder einstellen – selbst wenn es dieser Person seit einiger Zeit gut ginge. |
| 7 | Most people think less of a person who has attempted suicide. | Die meisten Menschen denken schlecht von einer Person, die einen Suizidversuch unternahm. | Most people think badly of a person, who attempted suicide. | Die meisten Menschen haben keine gute Meinung von einer Person, die einen Suizidversuch unternahm. |
| 8 | Most employers will hire a person who has attempted suicide if he or she is qualified for the job. | Die meisten Arbeitgeber werden eine Person, die einen Suizidversuch unternahm, einstellen, wenn sie für den Job qualifiziert ist. | Most employers will hire a person who attemtped suicide, if she is qualified for that job. | Die meisten Arbeitgeber werden eine Person, die einen Suizidversuch unternahm, einstellen, wenn sie für den Job qualifiziert ist. |
| 9 | Most employers will pass over the application of a person who has attempted suicide in favor of another applicant. | Die meisten Arbeitgeber werden die Bewerbung einer Person, die einen Suizidversuch unternahm, zugunsten eines anderen Bewerbers übergehen. | Most employers will ignore an application of a person who attempted suicide in favor of a different applicant. | Die meisten Arbeitgeber werden die Bewerbung einer Person, die einen Suizidversuch unternahm, zugunsten einer_s anderen Bewerbers_in übergehen. |
| 10 | Most people in my community would treat a person who has attempted suicide just as they would treat anyone. | Die meisten Menschen in meiner Gemeinde würden eine Person, die einen Suizidversuch unternahm, genauso so behandeln wie jeden anderen auch. | Most people in my community would treat a person who attempted suicide just as everyone else. | Die meisten Menschen in meiner Gemeinde würden eine Person, die einen Suizidversuch unternahm, genauso so behandeln wie jede_n andere_n auch. |
| 11 | Most women/men would be reluctant to date a person who has attempted suicide. | Die meisten Frauen/Männer würden nur widerwillig mit einer Person ausgehen, die einen Suizidversuch unternahm. | Most women/men would only reluctantly date a person who attempted suicide. | Die meisten Frauen/Männer würden nur widerwillig mit einer Person ausgehen, die einen Suizidversuch unternahm. |
| 12 | Once they know a person is a person who has attempted suicide, most people will take his/her opinion less seriously. | Sobald sie wissen, dass eine Person einen Suizidversuch unternahm, würden die meisten Menschen ihre Meinung weniger ernst nehmen. | As soon as people knew of a person, that she attempted suicide, they would take her opinion less seriously. | Sobald sie wissen, dass eine Person einen Suizidversuch unternahm, würden die meisten Menschen ihre Meinung weniger ernst nehmen. |
| 13 | Most people think that a person who has attempted suicide has a mental illness. | Die meisten Menschen denken, dass eine Person, die einen Suizidversuch unternahm, eine psychische Erkrankung hat. | Most people think that a person who attempted suicide suffers from mental illness. | Die meisten Menschen denken, dass eine Person, die einen Suizidversuch unternahm, eine psychische Erkrankung hat. |

**STOSASS**

| **Nr** | **Originalitem** | **Deutsche Übersetzung (Ulrike Maaß)** | **Englische Rückübersetzung** | **Revision der deutschen Items nach englischer Rückübersetzung** |
| --- | --- | --- | --- | --- |
| 1 | Most people would willingly accept a relative or a friend of a person who committed suicide as a close friend. | Die meisten Menschen würden bereitwillig eine Person, deren Angehöriger oder Freund Selbstmord beging, als einen engen Freund annehmen. | Most people would readily accept a person whose relative or friend committed suicide as a close friend. | Die meisten Menschen würden bereitwillig eine Person, deren Angehöriger_e oder Freund_in Selbstmord beging, als enge_n Freund_in akzeptieren. |
| 2a | People believe that a person who committed suicide was just as intelligent as the average person. | Menschen glauben, dass eine Person, die Selbstmord beging, genauso intelligent war wie der Durchschnittsmensch. | Most people think that a person who committed suicide was as intellient as the average person. | Menschen glauben, dass eine Person, die Selbstmord beging, genauso intelligent war wie der Durchschnittsmensch. |
| 2b | Most people believe that a relative or a friend of a person who committed suicide is just as intelligent as the average person. | Die meisten Menschen glauben, dass eine Person, deren Angehöriger oder Freund Selbstmord beging, genauso intelligent ist wie der Durchschnittsmensch. | Most people think that a person whose relative or friend committed suicide is just intelligent as the average person. | Die meisten Menschen glauben, dass eine Person, deren Angehörige_r oder Freund_in Selbstmord beging, genauso intelligent ist wie der Durchschnittsmensch. |
| 3a | Most people believe that a person who committed suicide was just as trustworthy as the average person | Die meisten Menschen glauben, dass eine Person, die Selbstmord beging, genauso vertrauenswürdig war wie der Durchschnittsmensch. | Most people think that a person who committed suicide, was just a trustworthy as the average person. | Die meisten Menschen glauben, dass eine Person, die Selbstmord beging, genauso vertrauenswürdig war wie der Durchschnittsmensch. |
| 3b | Most people believe that a relative or a friend of a person who committed suicide is just as trustworthy as the average person. | Die meisten Menschen glauben, dass eine Person, deren Angehöriger oder Freund Selbstmord beging, genauso vertrauenswürdig ist wie der Durchschnittsmensch. | Most people think that a person whose relative or friend committed suicide is as trustworthy as the average person. | Die meisten Menschen glauben, dass eine Person, deren Angehörige_r oder Freund_in Selbstmord beging, genauso vertrauenswürdig ist wie der Durchschnittsmensch. |
| 4 | Most people would accept a relative or a friend of a person who committed suicide as a teacher of young children in a public school. | Die meisten Menschen würden eine Person, deren Angehöriger oder Freund Selbstmord beging, als Lehrer an einer Schule für jüngere Kinder akzeptieren. | Most people would accept a person whose relative or friend committed suicide as a school teacher for younger children. | Die meisten Menschen würden eine Person, deren Angehörige_r oder Freund_in Selbstmord beging, als Grundschullehrer_in akzeptieren. |
| 5 | Most people feel that suicide is a sign of personal failure. | Die meisten Menschen finden, dass Selbstmord ein Zeichen für persönliches Versagen ist. | Most people think that suicide is a sign of personal failure. | Die meisten Menschen finden, dass Selbstmord ein Zeichen für persönliches Versagen ist. |
| 6 | Most people would not hire a relative or a friend of a person who committed suicide to take care of their children, even if he/she is healthy. | Die meisten Menschen würden keine Person, deren Angehöriger oder Freund Selbstmord beging, für die Versorgung ihrer Kinder einstellen – auch nicht, wenn er/sie gesund ist. | Most people wouldn't hire a person whose relative or friend committed suicide, as a caretaker for their children, not even if she was healthy/remitted. | Die meisten Menschen würden eine Person, deren Angehörige_r oder Freund_in Selbstmord beging, nicht für die Versorgung ihrer Kinder einstellen – selbst, wenn sie gesund ist. |
| 7a | Most people think less of a person who committed suicide. | Die meisten Menschen denken schlecht von einer Person, die Selbstmord beging. | Most people think badly of a person who committed suicide. | Die meisten Menschen haben keine gute Meinung von einer Person, die Selbstmord beging. |
| 7b | Most people think less of a relative or a friend of a person who committed suicide. | Die meisten Menschen denken schlecht von einer Person, deren Angehörigen oder Freund Selbstmord beging. | Most people think badly of a person whose relative or friend committed suicide. | Die meisten Menschen haben keine gute Meinung von einer Person, deren Angehörige_r oder Freund_in Selbstmord beging. |
| 8 | Most employers will hire a relative or a friend of a person who committed suicide if he or she is qualified for the job. | Die meisten Arbeitgeber werde eine Person, deren Angehöriger oder freund Selbstmord beging, einstellen, wenn sie/er für den Job qualifiziert ist. | Most employers will hire a person whose relative of friend committed suicide, if she is qualified for the job. | Die meisten Arbeitgeber werde eine Person, deren Angehörige_r oder Freund_in Selbstmord beging, einstellen, wenn sie für den Job qualifiziert ist. |
| 9 | Most employers will pass over the application of a relative or a friend of a person who committed suicide in favor of another applicant. | Die meisten Arbeitgeber werden die Bewerbung einer Person, deren Angehöriger oder Freund Selbstmord beging, zugunsten eines anderen Bewerbers übergehen. | Most employers will ignore the application of a person whose relative of friend committed suicide in favor of a different applicant. | Die meisten Arbeitgeber werden die Bewerbung einer Person, deren Angehörige_r oder Freund_in Selbstmord beging, zugunsten eines/r anderen Bewerbers_in übergehen. |
| 10 | Most people in my community would treat a relative or a friend of a person who committed suicide just as they would treat anyone. | Die meisten Menschen in meiner Gemeinde würden eine Person, deren Angehöriger oder Freund Selbstmord beging, genauso so behandeln wie jeden anderen auch. | Most people of my community would treat a person whose relative or friend committed suicide just as everyone else. | Die meisten Menschen in meiner Gemeinde würden eine Person, deren Angehörige_r oder Freund_in Selbstmord beging, genauso so behandeln wie jeden anderen auch. |
| 11 | Most women/men would be reluctant to date a relative or a friend of a person who committed suicide. | Die meisten Frauen/Männer würden nur widerwillig mit einer Person ausgehen, deren Angehöriger oder Freund Selbstmord beging. | Most women/men would only reluctantly date a person whose relative or friend committed suicide. | Die meisten Frauen/Männer würden nur widerwillig mit einer Person ausgehen, deren Angehörige_r oder Freund_in Selbstmord beging. |
| 12 | Once they know a person is a relative or a friend of a person who committed suicide, most people will take his/her opinion less seriously. | Sobald sie wissen, dass eine Person ein Angehöriger oder Freund einer Person ist, die Selbstmord beging, würden die meisten Menschen seine/ihre Meinung weniger ernst nehmen. | As soon as they knew, that a person is a relative of friend of a person who committed suicide, most people would take that person's opinion less seriously. | Sobald sie wissen, dass eine Person ein_e Angehörige_r oder Freund_in einer Person ist, die Selbstmord beging, würden die meisten Menschen seine/ihre Meinung weniger ernst nehmen. |
| 13a | Most people think that a person who committed suicide had a mental illness. | Die meisten Menschen denken, dass eine Person, die Selbstmord beging, eine psychische Erkrankung hatte. | Most people think, that a person who committed suicide, suffered from a mental illness. | Die meisten Menschen denken, dass eine Person, die Selbstmord beging, eine psychische Erkrankung hatte. |
| 13b | Most people think that a relative or a friend of a person who committed suicide has a mental illness. | Die meisten Menschen denken, dass eine Person, deren Angehöriger oder Freund Selbstmord beging, eine psychische Erkrankung hat. | Most people think, that a person whose relative or friend committed suicide, suffers from a mental illness. | Die meisten Menschen denken, dass eine Person, deren Angehörige_r oder Freund_in Selbstmord beging, eine psychische Erkrankung hat. |
