# Supplementary material for: Online-group intervention after suicide bereavement through the use of webinars: study protocol for a randomized controlled trial
Source: Trials. 2020 Jan 8;21:45. doi: 10.1186/s13063-019-3891-5 (PMC6951011; doi:10.1186/s13063-019-3891-5)
Supplement: Supplementary file 5 — Additional file 5. Questionnaire on Police Delivery of Death News. [file 13063_2019_3891_MOESM5_ESM.docx]

Appendix F

Trial Registration – German Clinical Trial Register (DRKS)

DRKS-ID:  DRKS00014426

**Trial Description**

**Title**

Web-based preventive group intervention for persons bereaved by suicide: A randomized control group trial

**Trial Acronym**

GROUP-CARE

**URL of the Trial**

<https://www.medicalschool-berlin.de/forschung/psychologie/online-intervention/group-care/>

**Brief Summary in Lay Language**

In Germany alone, there are approximately 10,000 people who die from suicide every year. Studies suggest that at least 6 to 10 close people are directly affected by the suicide struggling with the psychological consequences. Since 1989, the WHO describes persons bereaved by suicide as a high-risk group for suicide. Hence, the aftercare of this group is an integral part of national and international suicide prevention. The present web-based prevention project GROUP-CARE is a low-threshold cognitive-behavioral prevention program that takes into account the specific situation of persons bereaved by suicide. The online prevention program is geographically independent and takes place in a group setting using the form of a webinar. It is directed at adults between 18 and 75 years bereaved by suicide.

**Brief Summary in Scientific Language**

Objectives: Numerous population-based representative studies have shown increased survivor mortality after suicide compared to control groups. Therefore, the aim of the present study is to reduce the suicide risk for those bereaved by suicide using a web-based, cognitive-behavioral prevention program (GROUP-CARE). GROUP-CARE is a structured 12-week online intervention that brings together 6-8 people once a week (in the form of an "e-learning tool") Under professional guidance (therapist, self-help group leader) the group discusses topics that are related to mourning (e.g., guilt, shame, stigmatization, own suicidal tendencies, future perspectives, etc.).

Method: We chose a randomized-controlled design with a treatment and waiting group (each with about 36 participants) to examine the effectiveness of the program. We used different measurements (e.g., to assess suicidal behavior, depression, PTSD, cognitions) at the macro level (pre, middle, post, follow-up) as well as at the micro level after each therapy session (assessment of depression, suicidality, and group cohesion).

Research questions and hypotheses: It is expected that the suicidality and the mourning-related symptoms will be significantly reduced by GROUP-CARE. In addition, we will be able to identify the processes (e.g., stigma experience, guilt) that are significantly related to the mourners' psychopathology. Further, the predictors (such as age, severity of symptoms) of overall treatment success will be identified. Last but not least, we will examine the associations between the symptoms of mourning or trauma and the relationship to the deceased.

Discussion: The usefulness and necessity of the specific prevention intervention for survivors is discussed. In addition, we will present ideas for future research projects.

**Keywords**

Prevention, suicide, survivors, bereaved by suicide, online therapy, internet, intervention, grief, randomized control study, cognitive behavioural therapy

**Organizational Data**

- DRKS-ID:  DRKS00014426
- Date of Registration in DRKS:  2018/04/12
- Date of Registration in Partner Registry or other Primary Registry:  [---]*
- Investigator Sponsored/Initiated Trial (IST/IIT):  yes
- Date of (leading) Ethics Committee Application: 2018/02/24
- Date of (leading) Ethics Committee Approval: 2018/03/03
- Ethics Approval/Approval of the Ethics Committee:  Approved
- (leading) Ethics Committee No.:  MSB-2018/11, MSH Medical School Hamburg

**Secondary IDs**

- [---]*

Health Condition or Problem studied

- Suicidality
- bereavement, grief

**Interventions/Observational Groups**

- Arm 1:   Treatment group: receives online group therapy immediately after registration, based on cognitive-behavioral principles (psychoeducation, cognitive restructuring, dealing with difficult emotions such as guilt, shame, suicidality)
  Period: 12 weeks, once a week
- Arm 2:   Waiting group: receives online group therapy 12 weeks after registration;
  Period: 12 weeks, once a week

**Characteristics**

- Study Type:  Interventional
- Study Type Non-Interventional:  [---]*
- Allocation:  Randomized controlled trial
- Blinding:  Open (masking not used)
- Who is blinded:  [---]*
- Control:  Control group receives no treatment, Other
- Purpose:  Prevention
- Assignment:  Parallel
- Phase:  N/A
- Off-label Drug use:  N/A

**Primary Outcome**

We expect a significant reduction in suicidality immediately after completion of the treatment, as well as three or six months later.
The questionnaire is provided in an online survey.

**Measurements to assess suicidality:**

- Revised Acquired Capability of Suicide Scale (ACSS-FAD, Spangenberg et al., 2014)
- Beck Depression Inventory (BDI-II, Hautzinger et al., 2006)
- Beck Scale for Suicide Ideation (Beck, Steer, & Ranieri, 1988)
- Revised Acquired Capability of Suicide Scale (ACSS-FAD, Spangenberg et al., 2016)

**Secondary Outcome**

1. General Process of Symptom Assessment

There are several measurement points:

- T1 = initial diagnostics
- T2 = middle of therapy
- T3 = end of therapy
- T4 = follow-up examination after 3 months
- T5 = follow-up examination after 6 months

1. Status Diagnosis
   The following questionnaire are collected in the initial diagnosis (T1):

- Socio-demographic questions
- Screening for Complex Posttraumatic Stress Disorder (SkPTBS, Dorr, Sack & Bengel, 2018)

After the initial diagnosis (T1) a personal telephone interview is carried out. In this, the following instruments will be used

- SKID-I: Major depression (Wittchen et al, 1997)
- SKID-II: Borderline disorder (Wittchen et al., 1997)
- Suicide Risk Assessment Interview (SRAI; Arnoldi et al., 2000; Knaevelsrud, 2005)
- Suicide Behaviors Questionnaire Revised (Glaesmer et al., 2017; translated by Wagner et al., 2013)
- Dutch Screening Device for Psychotic Disorders (SDPD, Lange et al., 2000, Knaevelsrud, 2005)

1. Target Criteria: Assessment During the Treatment
   1. Changes in the Course of Treatment:

- Changes in the extent of general psychopathology, stigmatization experience, grief reaction, perception of social support and hopelessness:
- Health questionnaire for patients, short form (PHQ, Löwe et al., 2002)
- Berlin Social Support Scales (BSSS, Schwarzer & Schulz, 2000)
- Stigma of Suicide and Suicide Survivor Scale (STOSASS, Scocco et al., 2012)
- Grief Experience Questionnaire (GEQ; Bailley et al., 2000; Barrett & Scott Original, 1989)
- Posttraumatic cognition inventory (PTCI; Müller et al., 2010, Foa et al., 1999, original translation by Ehlers & Boos, 2000)
- Scales for Hopelessness (H-scales, Krampen, 1994)
- Aquired Capability of Suicide Scale, Revised (ACSS-FAD, Spangenberg et al., 2014)
- Inventory of Complicated Grief (ICG, Prigerson, Maciejewski et al., 1995, German translation by Lumbeck, Brandstätter, & Geissner, 2012)
- Impact of Event Scale (IES, Maercker & Schützwohl, 1998)
- Beck Depression Inventory (BDI-II; Hautzinger et al., 2006)
  1. Changes in the Course of Treatment Sessions
     In addition, there will be measurements after each therapy session (regarding the perception of the therapy session). The following instruments will be used:
- Health questionnaire for patients (PHQ-D, Löwe, Spitzer, et al., 2002)
- "suicide item" of BDI-II (Hautzinger et al., 2006)
- Group questionnaire (GQ-D; Bormann, Burlingame, & Strauss, 2011)

**Countries of Recruitment**

- DE:   Germany

**Locations of Recruitment**

- Medical School Berlin,  Berlin
- Bundesverband Verwaiste Eltern und trauernde Geschwister e.V.
- Angehörige um Suizid e.V.
- Internet

**Recruitment**

- Planned/Actual:  Actual
- (Anticipated or Actual) Date of First Enrollment:  2018/06/01
- Target Sample Size:  72
- Monocenter/Multicenter trial:  Multicenter trial
- National/International:  National

**Inclusion Criteria**

- Gender:  Both, male and female
- Minimum Age:  18   Years
- Maximum Age:  75   Years

**Additional Inclusion Criteria**

- experienced loss of the suicide of a close person
- Men and women
- Adults between 18 and 75 years
- Access to the Internet
- German language skills
- signed consent form
- Currently not in psychotherapy

**Exclusion Criteria**

1. Acute Suicidality: Suicidality is assessed in the telephone interview using the Suicide Risk Assessment Interview (SRAI; Arnoldi, Van de Ven, Schrieken, & Lange, 2000; Knaevelsrud, 2005). If potential participants express acute suicidality, they will be excluded from the treatment. In addition, the "suicide item" of the Beck-Depression-Inventaroy (BDI-II; Hautzinger et al., 2006) is considered separately in the weekly after-session-measurements. The answer options of the item are: 1) I do not think about doing anything to me; 2) I sometimes think of suicide but would not do it; 3) I would like to kill myself; 4) I would kill myself if I had the opportunity. If participants indicate any other option than option 1), a telephone appointment will be arranged and a separate suicide screening will be conducted, which will check the concreteness and quality of the suicidal thoughts.
2. Increased Depressiveness: Participants who have a BDI-II score of > 35 are excluded from the treatment.
3. Psychotic Experience: Participants with a cut-off score > 13 in the Dutch Screening Device for Psychotic Disorder (SDPD; Knaevelsrud, 2005; Lange, Schrieken, Blankers, Van de Ven, & Slot, 2000) are excluded from the study.
4. Increased Alcohol, Drug, or Substance Use: Measured by the biographical questionnaire and the Structured Clinical Interview for DSM-IV Achse I (SKID-I; Wittchen, Zaudig, & Fydrich, 1997).
5. Persons already in psychotherapeutic treatment: Measured using the biographical questionnaire.
6. Borderline personality disorder and/or self-injurious behavior: Measured by the Structured Clinical Interview for DSM-IV Axis II (SKID-II; Wittchen et al., 1997)
7. Bipolar disorder: Measured by the SKID-I.

**Addresses**

***Primary Sponsor***

Medical School Berlin

Ms.  Prof.  Birgit  Wagner

Calandrellistr. 1-9

12247  Berlin

Germany

Telephone:  +49 30 / 76 68 37 5 -801

Fax:  +49 30 / 76 68 37 5 -619

E-mail:  [birgit.wagner at medicalschool-berlin.de](mailto:birgit.wagner@medicalschool-berlin.de)

URL:  <https://www.medicalschool-berlin.de/hochschule/unser-team/team-fakultaet-naturwissenschaften/professoren/prof-dr-habil-birgit-wagner/>

***Collaborator, Other Address***

AGUS Angehörige um Suizid e.V.

Mr Jörg  Schmidt

Cottenbacher Str. 4

95445  Bayreuth

Telephone:  09211500380

Fax:  09211500379

E-mail:  [kontakt at agus-selbsthilfe.de](mailto:kontakt@agus-selbsthilfe.de)

***Collaborator, Other Address***

Bundesverband Verwaiste Eltern und trauernde Geschwister in Deutschland e.V.

Ms Petra  Hohn

Roßplatz 8a

04103  Leipzig

Telephone:  0341-9468884

Fax:  0341-9023490

E-mail:  [kontakt at veid.de](mailto:kontakt@veid.de)

URL:  [http://www.veid.de](http://www.veid.de)

***Contact for Scientific Queries***

Medical School Berlin

Professor  Birgit  Wagner

Calandrellistr. 1-9

12247  Berlin

Germany

Telephone:  +49 30 / 76 68 37 5 -801

Fax:  [---]*

E-mail:  [birgit.wagner at medicalschool-berlin.de](mailto:birgit.wagner@medicalschool-berlin.de)

URL:  <https://www.medicalschool-berlin.de/hochschule/unser-team/team-fakultaet-naturwissenschaften/professoren/prof-dr-habil-birgit-wagner/>

***Contact for Public Queries***

MSB Medical School Berlin

Ms  Laura  Hofmann

Calandrellistr. 1-9

12247  Berlin

Germany

Telephone:  +49 30 / 76 68 37 5 -830

Fax:  [---]*

E-mail:  [laura.hofmann at medicalschool-berlin.de](mailto:laura.hofmann@medicalschool-berlin.de)

***(leading) Ethics Committee***

MSH Medical School Hamburg

Am Kaiserkai 1

20457 Hamburg

Germany

URL: <https://www.medicalschool-hamburg.de/forschung/qualitaetssicherung/>

***Sources of Monetary or Material Support***

Public funding institutions financed by tax money/Government funding body (German Research Foundation (DFG), Federal Ministry of Education and Research (BMBF), etc.)

Bundesministerium für Gesundheit

Friedrichstr. 108

10117  Berlin

Germany

Telephone:  030/18441-0

Fax:  [---]*

E-mail:  [poststelle at bmg.bund.de](mailto:poststelle@bmg.bund(dot)de)

**Status**

- Recruitment Status:  Recruiting ongoing
- Reason, if „Recruitment stopped after recruiting started“ or „Recruiting withdrawn before recruiting started“: [---]*
- Reason, if Reason for Recruiting Stop “Other”: [---]*
- Study Closing (LPLV):  [---]*
- Number of Participants in Germany after Recruiting complete: [---]*
- Total Number of Participants (all Sites worldwide) after Recruiting complete: [---]*

**Trial Publications, Results and other Documents**

- Approval of ethics comm. (mandatory for transfer to Studybox):   [Ethikbescheid](https://www.drks.de/ui_data_web/AttachmentDownstreamServlet?ID=6a648143-8873-45bf-b112-fde4732f3f68&LOCALE=en&FILENAME=BR%20Ethikbescheid%20Wagner%20GroupCare%202018-03-03.pdf)
